# Tumor Purity Coexpressed Genes Related to Immune Microenvironment and Clinical Outcomes of Lung Adenocarcinoma

**DOI:** 10.1155/2021/9548648

**Published:** 2021-06-14

**Authors:** Ming Bai, Qi Pan, Chen Sun

**Affiliations:** ^1^Second Department of Medical Oncology, The First Hospital of China Medical University, Shenyang 110001, China; ^2^Department of Hepatobiliary Surgery and Organ Transplantation, The First Hospital of China Medical University, Shenyang 110001, China; ^3^Department of Radiology, Shengjing Hospital of China Medical University, Shenyang 110001, China

## Abstract

**Purpose:**

Lung cancer tissue includes tumor tissue, stromal cells, immune cells, and epithelial cells. These nontumor cells dilute the tumor purity in lung cancer tissues. Tumor purity plays an essential role in the immune response to lung cancer. At present, the biological processes related to the purity of lung cancer tumors remains unclear.

**Methods:**

We measured tumor purity in 486 lung carcinoma tissues from TCGA-LUAD FPKM by using the “estimate” R package. Lung carcinoma tumor mutation burden was calculated by analyzing TCGA single nucleotide polymorphism data. The immune cell proportion was also evacuated via the CIBERSORT method. Lung carcinoma samples with *P* < 0.05 were considered significant. Based on the tumor purity and lung carcinoma gene matrix, we performed weighted gene coexpression network analysis (WGCNA), and the tumor purity-related module was identified. Then, we analyzed the functions of the factors involved in the module. We screened the coexpressed factors related to clinical outcome and immunophenotype. Finally, expression levels of these factors were measured at tissue and single-cell levels.

**Results:**

A lung cancer tumor purity correlated coexpression network was determined. Five coexpressed genes (CD4, CD53, EVI2B, PLEK, and SASH3) were identified as tumor purity coexpressed genes that negatively correlated with tumor purity. Because the factors in the coexpression network often participate in similar biological processes, we found that CD4, CD53, EVI2B, PLEK, and SASH3 were most related to positive regulation of cytokine production and interleukin−2 production through functional enrichment. In a clinical phenotype analysis, we found that these five factors can be used as independent prognostic risk factors. We found that these factors were significantly negatively correlated with tumor purity and positively correlated with the immune score in the immunophenotyping analysis. Using GSEA analysis, we found that the antigen processing and presentation pathway were related to the five tumor coexpressed genes mentioned above. SASH3 and CD53 were used to conduct a prognostic model based on the interaction analysis of the Support Vector Machine and the Least Absolute Shrinkage and Selection Operator. SASH3 was verified to be related to CD8A using a single-cell analysis.

**Conclusion:**

Tumor purity-related coexpression factors in the tumor microenvironment have essential clinical, genomic, and biological significance in lung cancer. These coexpression factors (SASH3 and CD53) can be used to classify tumor purity phenotypes and to predict clinical outcomes.

## 1. Introduction

The structure of tumor tissue is complex. In addition to tumor cells, there are also other components such as stromal cells, inflammatory cells, vasculature, and the extracellular matrix [1].

Tumor microenvironment possesses complexity because of a mixture of growth-promoting and inhibiting growth factors, nutrients, chemokines, and other noncancer types, which interact with each other and associate with tumor growth, disease progression, drug resistance, and especially, infiltrating T lymphocytes and tumor growth [2]. Approximately 1.8 to 8 million people are diagnosed with lung cancer each year, and 1.6 to 6 million die from lung cancer. The 5-year survival rate of lung cancer is about 4–17% [3]. Although studies have revealed the mechanisms involved in cancer malignant characteristics and identified reasonable therapeutic targets [4], current clinical prediction and treatment outcome of lung cancer are not satisfactory [5]. We are now aware that lung cancer tissues are rich in nontumor cells, of which stromal cells significantly regulate tumor proliferation, invasion, and angiogenesis [6].

With the development of bioinformatics in recent years and the acquisition of open lung cancer cohorts, it becomes possible to evaluate tumor purity content in the samples according to the estimated infiltration of stromal and immune cells ESTIMATE algorithm [7]. In this study, we constructed a tumor purity coexpression network based on weighted gene coexpression network analysis (WGCNA) [8]. We explored the coexpression factors most related to tumor purity and related biological functions and demonstrated the most relevant biological functions and mechanism of action affecting tumor purity in the lung cancer tumor microenvironment.

## 2. Methods

### 2.1. Data Sources

The Cancer Genome Atlas- (TCGA-) LUAD FPKM data containing 486 cancer tissue samples were obtained (http://cancergenome.nih.gov/) [9]. GSE99254 [10] is a single-cell sequencing cohort with 14 samples and 12346 non-small-cell lung cancer cells and was obtained from the GEO (http://www.ncbi.nlm.nih.gov/geo/) database with the GPL16791 platform and GPL20301 platform. Meanwhile, GSE42127 [11] was also downloaded to verify the conclusion.

### 2.2. Tumor Purity Evaluation

Expression data (ESTIMATE) [7] were used to evaluate stromal and immune cells in malignant tumor tissues in this study and estimate the proportion of stromal and immune cells in the tumor microenvironment based on the gene matrix. Through ESTIMATE algorithm, tumor purity of each lung cancer sample in TCGA-LUAD was obtained. CIBERSORT algorithm [12, 13] is a method in order to evaluate the cell content in bulk tissue gene expression matrices. Immune cell infiltration levels were calculated based on the LM22 matrix and CIBERSORT algorithm, and samples with *P* < 0.05 were considered significant and taken into this study.

### 2.3. Tumor Purity-Related Coexpression Factors

Weighted Gene Coexpression Network Analysis (WGCNA) was demonstrated to determine tumor purity coexpressed genes in lung cancer. This method converted tumor purity coexpression correlations into weight values which determined the coexpression factors. As we know, the expression levels of genes were approximately the same as those possessing similar biological functions [14]. In this research, we set the soft threshold as 5, *R* square = 0.98, and the factors in the minimum module as 30. We uploaded tumor purity scores and immune cell proportions as phenotype files. In this manner, a cluster of tumor purity coexpression genes with similar biological function was determined via WGCNA [15].

### 2.4. Protein-Protein Network and Function Analysis

The encoding genes of tumor purity coexpression proteins were identified by the Pearson correlation coefficient >0.4. The coexpression modules of tumor purity were conducted by Cytoscape software. Meanwhile, the tumor purity coexpressed genes were enriched to explore their biological processes in the tumor microenvironment. The Database for Annotation, Visualization, and Integrated Discovery (DAVID, v6.8) is an online database which provides functional annotation analysis [16, 17]. The Kyoto Encyclopedia of Genes and Genomes (KEGG) [18] (https://www.genome.jp/kegg/) and Gene Ontology (GO) [19] (http://geneontology.org/) analysis were used to identify the biological function and related regulation pathways in each coexpression module.

### 2.5. Prognosis Model Based on LASSO and Support Vector Machine Methods

Univariate Cox regression analysis was performed for tumor purity coexpression genes, and the genes with *P* < 0.05 were taken into the feature selection. Subsequently, we screened the characteristic variables and constructed the prognostic model by observing LASSO regression analysis [20–22] and support vector machine (SVM-RFE algorithms).

### 2.6. Gene Set Enrichment Analysis (GSEA)

GSEA calculates the significance and consistency differences of a predefined dataset between two biological states [23]. The gene matrix in TCGA was divided into high- and low-expression groups, following the median expression level of lung cancer tumor purity-related genes. Through GSEA analysis, we obtained the related pathways of genes which were correlated with tumor purity and prognosis. These pathways are considered related to the immune microenvironment as well as clinical phenotypes.

### 2.7. Immune Correlation in Other Types of Cancer

The TIMER database (https://cistrome.shinyapps.io/timer/) [24, 25] was applied to show the correlations between SASH3 and immune cell proportion in 33 types of cancers. A Pearson correlation coefficient higher than 0.4 was considered significant.

### 2.8. Single-Cell Cohort Analysis

We found that the factor with the strongest negative correlation with tumor purity was CD8+ T lymphocytes. Therefore, we aimed to verify this relationship at the single-cell sequencing level. We obtained the GSE99254 single-cell cohort from the GEO database. The Seurat package was then used to filter and standardize the data [26]. Various cell subpopulations were obtained by the TSNE dimensionality reduction clustering method [27]. Finally, the SingleR package was used to annotate the cell types of these subpopulations [28]. We demonstrated the relationship between SASH3 and CD8+ T lymphocyte infiltration by labeling the relationship between SASH3 and CD8+ T lymphocyte infiltration.

### 2.9. Statistical Analysis

R 3.6.3 (https://www.r-project.org/) was carried out for statistical analysis. Student's *t*-tests are applied to show purity differences in various subgroups in the TCGA cohort. Coexpression coefficients of tumor purity protein encoding genes were evaluated based on the Pearson correlation.

## 3. Results

### 3.1. Tumor Purity Coexpression Network

**Figure 1:**
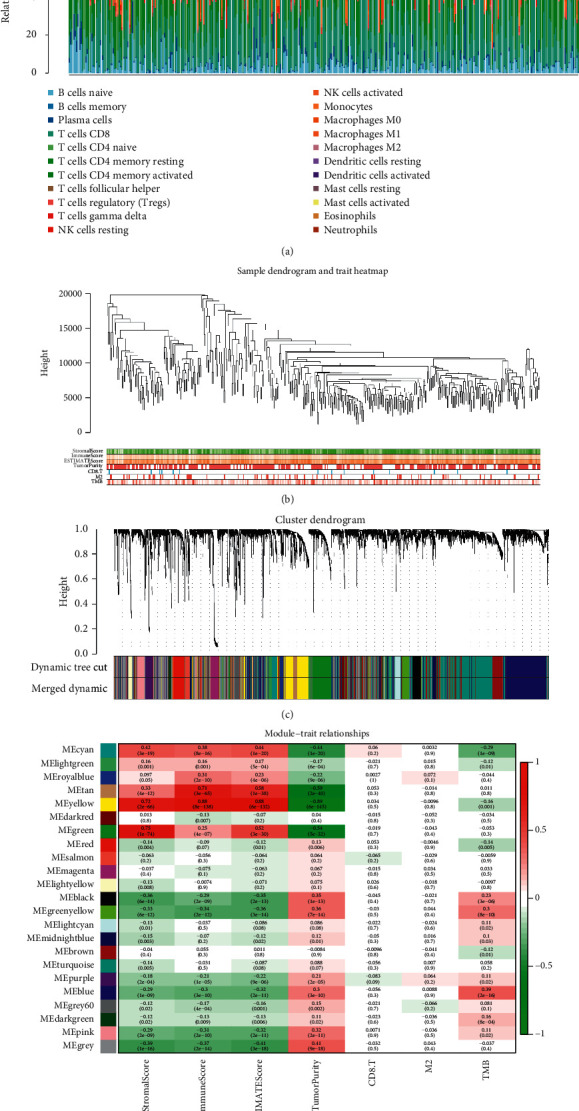
(a) The proportion of 22 kinds of immune cells in the tumor microenvironment of lung adenocarcinoma. (b) The hierarchical clustering tree was obtained by the dynamic mixing cutting method. (c) A total of 23 coexpression modules were obtained, in which each leaf represented a gene and each branch represented a coexpression module. (d) Correlation of different modules and various phenotypes. The yellow module had a strong positive correlation with the stromal score (Cor = 0.72; *P*=2*e* − 26), immune score (Cor = 0.88; *P*=8*e* − 138), and ESTIMATE score (Cor = 0.88; *P*=6*e* − 132). The negative correlation between the yellow module and tumor purity was strong (Cor = −0.89; *P*=6*e* − 145). The green module had a strong positive correlation with the stromal score (Cor = 0.75; *P*=1*e* − 74) and ESTIMATE score (Cor = 0.52; *P*=3*e* − 30). The negative correlation between the green module and tumor purity was strong (Cor = −0.54; *P*=5*e* − 32).

We obtained tumor purity and immune cell content of each person in TCGA-LUAD which is uploaded into Supplementary Table 1. The corresponding immune cell content in each sample is shown in Figure 1(a). The results demonstrated that CD8+ T lymphocytes and CD4+ cells had the highest content. Next, a dimension-reducing cluster was conducted (Figure 1(b)) for the samples of TCGA-LUAD using omics clustering. We obtained 23 coexpression networks (Figure 1(c)) through WGCNA, where each color represented one coexpression network. Furthermore, we examined the correlation between coexpression networks and tumor purity to identify the most relevant ones. The results elucidated that the yellow and green modules had the strongest correlation with tumor purity (Figure 1(d)). WGCNA results have been uploaded in Supplementary Table 2.

### 3.2. Protein-Protein Network and Function Enrichment

**Figure 2:**
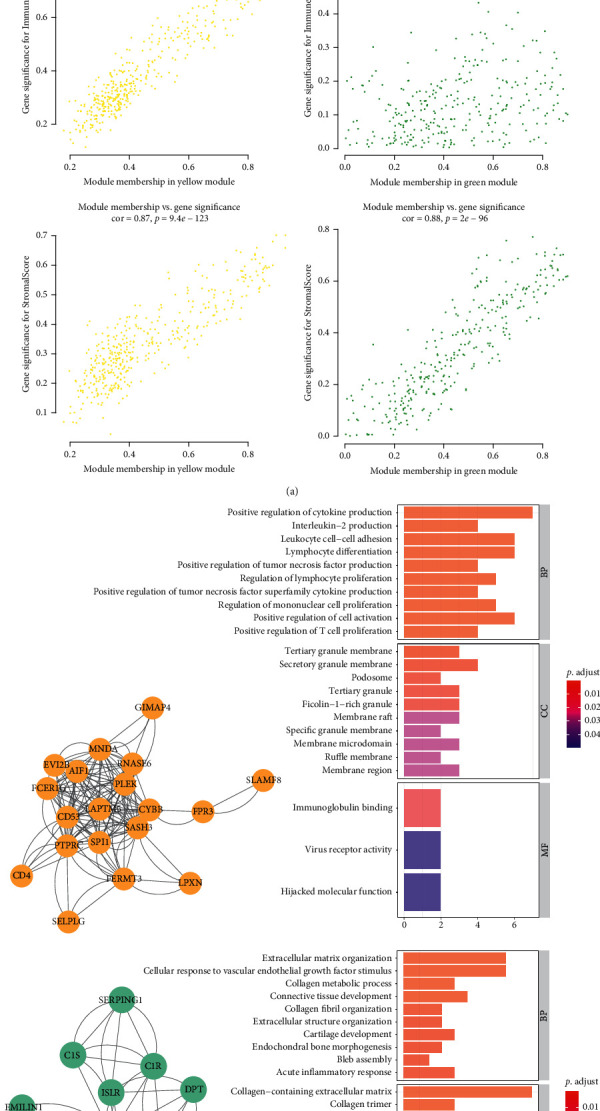
(a) The correlation between gene significance for tumor purity, immune score, and stromal score with module membership in the yellow or green module. (b) The PPI network was constructed using coexpressed genes of the yellow and green modules and the main enriched pathway of coexpressed genes. The yellow module was enriched in BP pathways, including positive regulation of cytokine production, leukocyte cell-cell differentiation, and lymphocyte differentiation. The green module was enriched in BP pathways, including cellular response to vascular endothelial growth factor stimulus and extracellular matrix organization.

We plotted a scatter plot of the correlation between tumor purity and coexpression modules in the yellow and green modules (Figure 2(a)). The results showed that the correlation between tumor purity and gene coexpression module in the yellow module was the most significant (COR = 0.96; *P*=*e* − 200), whereas the correlation in the green module was lower (COR = 0.73; *P*=3.5*e* − 35). Furthermore, GO enrichment analysis of genes in the yellow module suggested that positive regulation of chemokines and the generation of interleukin-2 were the most significant enriched pathways (Figure 2(b)), while genes in the green coexpression module are associated with the extracellular matrix. Thereby, the protein encoding genes in the yellow coexpression module were selected for subsequent analyses.

### 3.3. Clinical Phenotype and Immune Phenotype

**Figure 3:**
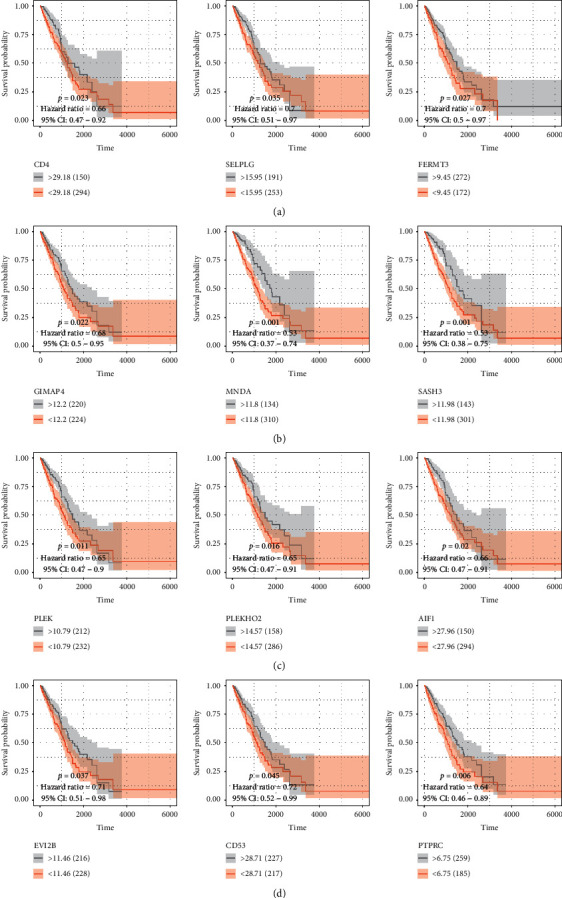
Survival analysis of CD4 (*P*=0.023; HR = 0.66), SELPLG (*P* = 0.035; HR = 0.7), FERMT3 (*P*=0.027; HR = 0.7), GIMAP4 (*P*=0.022; HR = 0.68), MNDA (*P*=0.001; HR = 0.53), SASH3 (*P*=0.001; HR = 0.53), PLEK (*P*=0.011; HR = 0.65), PLEKHO2 (*P*=0.016; HR = 0.65), AIF2 (*P*=0.02; HR = 0.66), EVI2B (*P*=0.037; HR = 0.71), CD53 (*P*=0.045; HR = 0.72), and PTPRC (*P*=0.006; HR = 0.64). All of the results were statistically significant.

**Figure 4:**
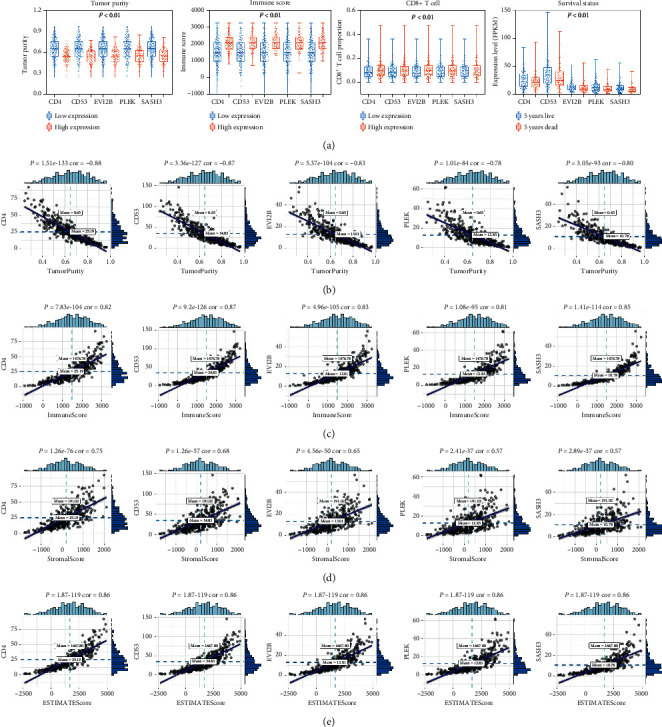
(a) Difference analysis of five essential genes in tumor purity, immune score, CD8+ T cell, and survival status. (b) Correlation analysis of five essential genes with tumor purity, (c) immune score, (d) stromal score, and (e) ESTIMATE score.

**Figure 5:**
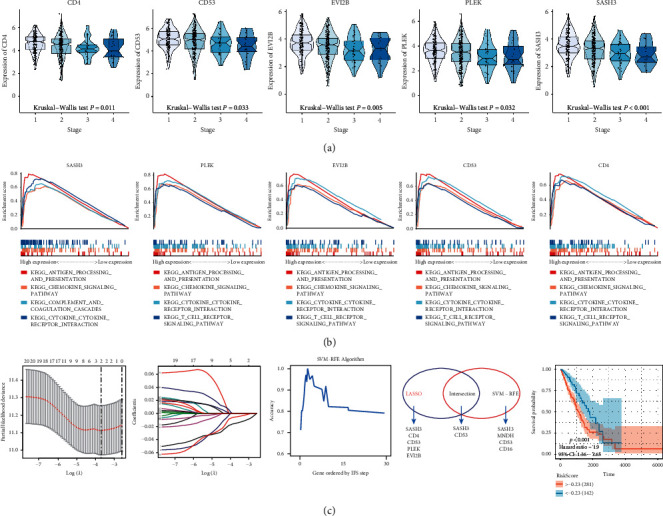
(a) Five essential genes can distinguish the different clinical stages of lung adenocarcinoma. (b) GSEA analysis of the five essential genes. The antigen processing and presentation pathway, chemokine signaling pathway, cytokine receptor interaction pathway, and T-cell receptor signaling pathway were related to the five tumor purity coexpression genes. (c) Combined with LASSO regression and support vector machine algorithm, SASH3 and CD53 were finally screened as prognostic genes of lung adenocarcinoma.

The survival analysis for factors in the yellow module that can be used as independent prognostic evaluation for overall survival is shown in Figure 3. We then performed clinical and immunophenotypic assessments of these factors (Figure 4). The results suggested that CD4, CD53, EVI2b, PLEK, and SASH3 correlated with tumor purity, immune score, CD8+ T cells, and clinical phenotypes (Figure 4(a)). Low expression level of these genes led to high tumor purity, low immune score, low CD8+ T lymphocyte content, and shorter 5-year survival. The scatter plots of correlations between CD4, CD53, EVI2b, PLEK, SASH3, and tumor environment score are shown in Figures 4(b)–4(e). Results shown in Figure 5(a) indicated negative correlations between the clinical stages and the expression of these five genes (Figure 5(a)).

### 3.4. GSEA Analysis

GSEA analysis elucidated that chemokine-chemokine receptor interaction and the T-cell receptor signaling pathway were enriched in the high expression group of factors in the prognosis model (Figure 5(b)). These pathways enhanced the immune response and showed antitumor immune response. These factors might reduce tumor purity of lung cancer by elevating lymphocyte proportion.

### 3.5. LASSO Regression and SVM

We incorporated the protein encoding genes in the yellow coexpression module into the LASSO regression model and identified five significant prognostic survival genes (SASH3, PLEK, EVI2B, CD53, and CD4). Simultaneously, the support vector machine method was used to screen the features of the abovementioned factors, and four feature variables (SASH3, MNDH, CD53, and CD16) were determined. We finally identified SASH3 and CD53 as tumor purity-related prognostic factors (Figure 5(c)). Risk score = −0.004*∗*CD53 – 0.014*∗*SASH3. We later found a significant survival difference in lung cancer patients in the TCGA-LUAD cohort between the two risk scores (HR = 1.9; *P* < 0.001).

### 3.6. SASH3 Related to CD8+ T Cell and Immunohistochemistry

**Figure 6:**
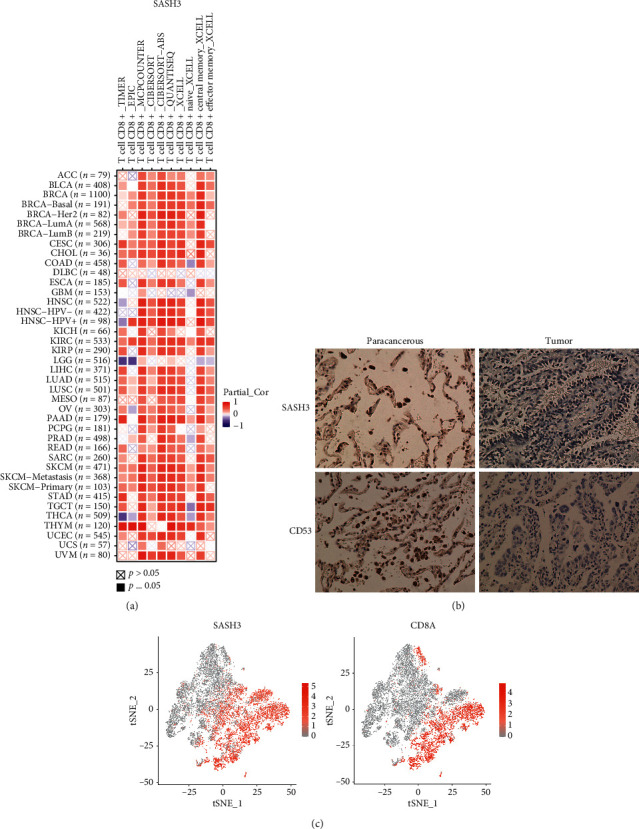
(a) In the pan-cancer spectrum, SASH3 is strongly correlated with CD8+ T cells. Red color means positive correlation, while purple means negative correlation. (b) Immunohistochemical expression of SASH3 and CD53 in lung adenocarcinoma and paracancerous tissues. (c) In single-cell sequencing cohort validation, clusters with high expression of SASH3 were similar to those with high expression of CD8+ T cells.

In the abovementioned study, we found that SASH3 was significantly negatively correlated with tumor purity but positively correlated with CD8+ T lymphocytes. To further verify this positive correlation, we verified this conclusion in 33 TCGA-type cancers. The results showed that SASH3 was positively correlated with the content of CD8+ T lymphocytes in lung cancer, glioma, liver cancer, and other cancers (Figure 6(a)). At the same time, we found that the staining strength of the SASH3 antibody in lung cancer tissues of China Medical University was higher in paracancerous tissues but relatively lower in tumor tissues (Figure 6(b)). Finally, we found that the distribution of SASH3 in the single-cell cohort was like that of the CD8+ T lymphocyte biomarker CD8A (Figure 6(c)). Finally, we added external queue validation to prove the correlation between SASH3 and CD8A in GSE42127 (Supplementary Figure 1).

## 4. Discussion

In this study, we first calculated the tumor purity of lung cancer tissue. Then, we established a coexpression network related to tumor purity of lung cancer, thereby obtaining the two modules with the highest correlation to tumor purity. A PPI network was established for critical genes in the module, five coexpressed genes were identified, and the enriched pathways were calculated. Then, two machine learning methods (LASSO regression and SVM) were used to establish the model. The intersection was taken to screen out that SASH3 and CD53 were tumor purity-related prognostic genes of lung cancer. Clinical phenotype and immune phenotype assessments of the coexpressed genes showed that SASH3 negatively correlated with tumor purity and positively correlated with CD8+ T lymphocytes. This result was verified by single-cell cohort sequencing, pan-cancer analysis, and immunohistochemistry.

T cells were dominant in lung cancer. CD4+ T cells (26%) were the most abundant T cell population, followed by CD8+ T cells (22%) [29]. CD4 encodes the CD4 membrane glycoprotein of T lymphocytes. The CD4 antigen and the T-cell receptor on the T lymphocyte work together to complete the antigen presentation and recognition [30]. Many scientists are interested in the role of CD4 immunity in the efficacy of PD-L1/PD-1 blocking therapy. Kagamu et al. found that immune monitoring of CD4+ T cells in peripheral blood predicted anti-PD-1 treatment responses in lung cancer patients [31]. Preclinical studies in patients and mouse models have demonstrated the importance of CD4 immunity for immunotherapy [32]. Patients who responded to treatment showed a high proportion of CD4+ T cells before treatment. These CD4+ T cells demonstrated proliferation at baseline and responded to PD-1 blockade [33]. These findings support the idea of using vaccination to enhance CD4+ neoantigen-specific T cells in antitumor immunity [34].

Antitumor immunity is determined by the presence of different immune cells in the tumor microenvironment (TME). Environmental signals transmitted through the plasma membrane determine whether immune cells are activated or suppressed. Tetrantin proteins are a significant component of the plasma membrane because they aggregate immune receptors, enzymes, and signaling molecules into the tetrantin reticulum [35]. CD53 is a four-transmembrane protein, mainly expressed in the myeloid lymphoid system [36]. Yunta and Lazo found that CD53 antigen stimulation may have a protective effect on programmed cell death. CD53 antigen interaction protects against the apoptotic response caused by serum deprivation and contributes to cell survival in the poorly vascularized region of the tumor mass [37]. CD53 is also essential for B-cell function because CD53 promotes BCR-dependent protein kinase C signaling, allowing it to phosphorylate its substrate [38].

SAM and SH3 domain containing 3 (SASH3) encoded proteins act as signal transduction proteins in lymphocytes [39]. Pleckstrin (PLEK) is a protein found in platelets and white blood cells that acts as a substrate for protein kinase C [40]. The ecotropic viral integration site 2B (EVI2B) gene was in the intron of the neurofibromatosis type 1 (NF1) gene and transcribed in the opposite direction to the NF1 gene [41]. Like the NF1 gene, EVI2B is involved in the differentiation of melanocytes and keratinocytes [42]. There is currently a lack of research on the relationship between EVI2B, SASH3, and PLEK with lung cancer. However, Huang et al. analyzed the genes of colorectal cancer patients with membrane array and direct sequencing and found that EVI2B may be a potential prognostic marker in CRC patients [43]. Other scholars found that there are mutations in EV2B in the mutation spectrum of breast cancer cell line NZBR under conditions of physiological oxygen concentrations [44].

Although this study integrated relatively multiple bioinformatics analysis and improved immunohistochemical experimental verification, there are some limitations. More large samples are needed to validate our results. In vitro and in vivo experiments should be conducted, and a feasibility study for clinical practice should be contemplated.

In conclusion, two coexpression factors (SASH3 and CD53) help classify tumor purity phenotypes and predict clinical phenotype in lung cancer with the chemokine signaling pathway. The mechanism might provide concepts to modify the curative effect in patients with high tumor purity.

## Data Availability

The datasets TCGA-BRCA for this study can be found in the The Cancer Genome Atlas (http://cancergenome.nih.gov/). The datasets GSE99254 in this study can be found in the GEO (http://www.ncbi.nlm.nih.gov/geo/).

## Supplementary Materials

Supplementary Materials Supplementary Table 1: the results of tumor purity. Supplementary Table 2: the results of WGCNA analysis. Supplementary Figure 1: the verification of the correlation between CD8A and SASH3 in the GEO database.
