# Supplementary figures and images for: Tumor Purity Coexpressed Genes Related to Immune Microenvironment and Clinical Outcomes of Lung Adenocarcinoma
Source: J Oncol. 2021 Jun 14;2021:9548648. doi: 10.1155/2021/9548648 (PMC8216812; doi:10.1155/2021/9548648)

GSE42127

$\log_e(S) = 12.38$ ,  $p = 1.24\text{e-}31$ ,  $\hat{\rho}_{\text{Spearman}} = 0.74$ ,  $\text{CI}_{95\%} [0.66, 0.80]$ ,  $n_{\text{pairs}} = 176$

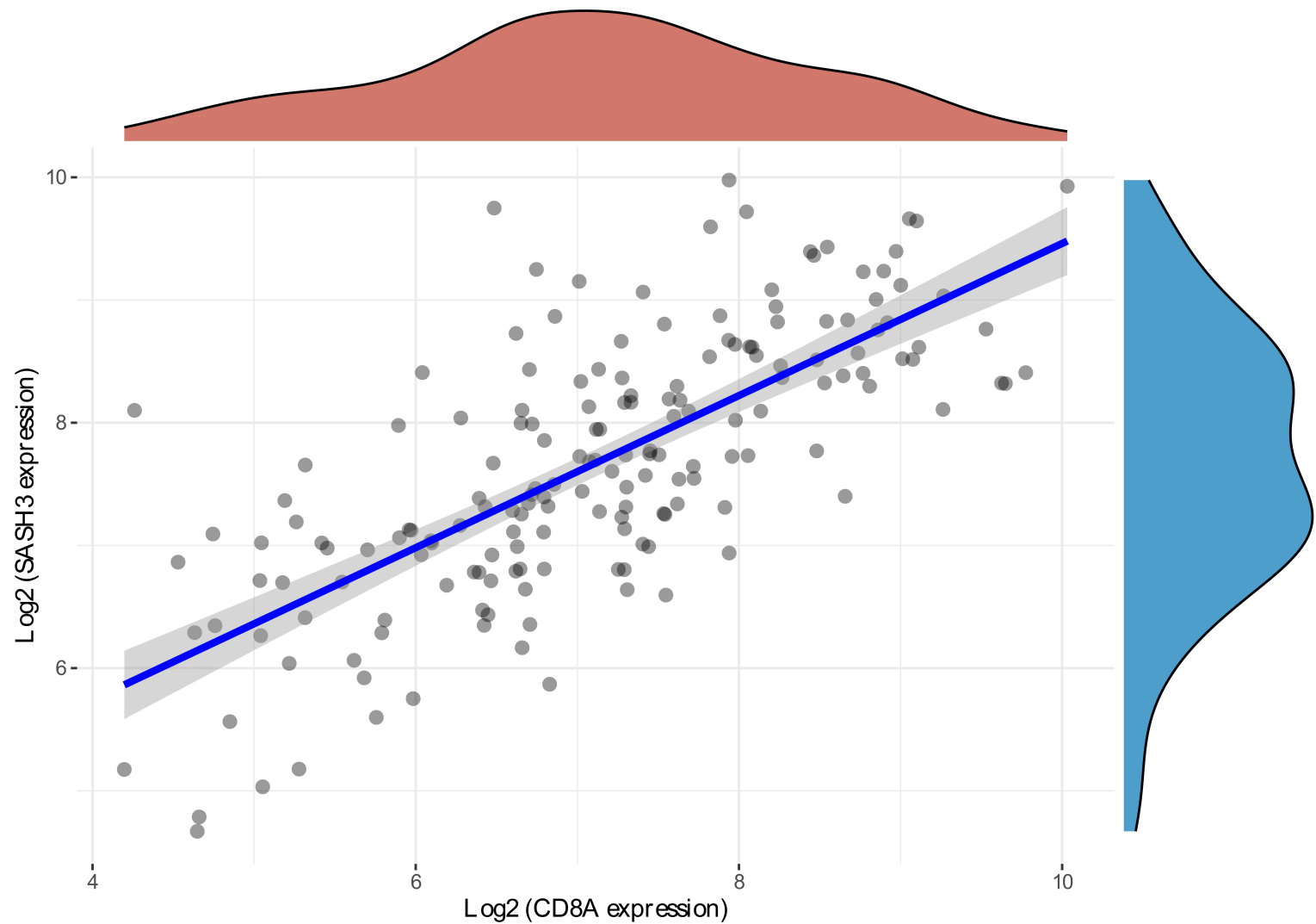

Supplement: Supplementary Materials — Supplementary Table 1: the results of tumor purity. Supplementary Table 2: the results of WGCNA analysis. Supplementary Figure 1: the verification of the correlation between CD8A and SASH3 in the GEO database. [file 9548648.f1.zip › 9548648.f1/Supplementary figure 1.pdf]
